# Compact Measurement of the Optical Power in High-Power LED Using a Light-Absorbent Thermal Sensor

**DOI:** 10.3390/s21144690

**Published:** 2021-07-08

**Authors:** You-Young Kim, Jae-Young Joo, Jong-Min Kim, Sun-Kyu Lee

**Affiliations:** 1 School of Mechanical Engineering, Gwangju Institute of Science and Technology, Gwangju 61005, Korea; youyoung@gm.gist.ac.kr (Y.-Y.K.); jongminkim@gm.gist.ac.kr (J.-M.K.); 2 Korea Photonics Technology Institute, Gwangju 61007, Korea; jyjoo@kopti.re.kr

**Keywords:** photo-thermal sensor, light absorption, high-power LED, surface temperature

## Abstract

LED (Light-Emitting Diode) presents advantages such as luminescence, reliability, durability compared with conventional lighting. It has been widely applied for life, healthcare, smart farm, industry, and lighting from indoor to the automotive headlamp. However, the LED is vulnerable to thermal damage originated from the high junction temperature, especially in high power applications. Hence, it requires precise qualification on the optical power and the junction temperature from the pilot line to secure reliability. In this study, the photo-thermal sensor is proposed by employing a sheet-type thermocouple composed of photo-absorbent metal film and thermocouple. This sensor aims low-cost qualification in pilot line for high-power luminous devices and optical monitoring of costly luminaire such as automobile LED headlamp. The sensor is designed to detect the increased temperature response of LED hot spots from the transferred thermal power and absorbed optical power. The temperature response of each sheet-type thermocouple is utilized as a signal output of the absorbed optical power and hot spot temperature based on the introduced sensor equation. The proposed thermal sensor is evaluated by comparing the experiment with the measured reference value from the integrating sphere and the attached thermocouple at a junction. The experiment result reveals 3% of the maximum error for the optical power of 645 mW.

## 1. Introduction

The LED has significantly been developed based on its advantages such as small volume, impact resistance, high reliability, long life, and low power consumption, and it became a remarkable measure for convenient use, energy-saving, and environmental protection. Recently, the high-power LED industry has widened its application from indoor lighting to the health care, display, architectural industry, and even automotive headlights; however, thermal reliability of high-power LED is required more than ever due to the high integration and miniaturization of the package. The optical power and the hot spot temperature of the LED must be qualified in the pilot line and tested robustly to secure the functions without failure. In terms of its optical performance, however, the total optical power must be increased with higher input power and integration of the light source, which results in instability of the thermal system and reduction in lifetime and lighting performance [1]. Recent studies have been focused on the thermal management of the LED application. Ha et al. [2] pointed out that the performance of high-power LEDs strongly depended on the junction temperature, and the operation at high junction temperature causes degradation of both optical power and lifetime. Wu et al. [3] estimated the dissipation of heat in the high-power COB (Chip-on-Board) LEDs by evaluating the junction temperature and thermal resistance on different gaps.

**Figure 1:**
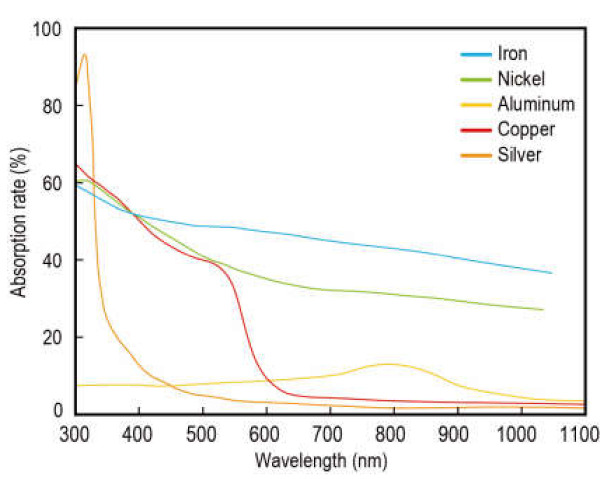
Optical absorption ratio of general metal materials.

The hot spot temperature was also compared with an infrared thermal imaging camera measurement, but the result is still questionable due to the additional light loss on the phosphor layer, which can cause measuring error. Christensen and Zhao present a thermal network model integrated with a sub-model of a structure of LED in finite element to predict the die temperatures [4,5]. Cheng et al. [6] present a thermal management method for an array of the high-power LED based on a multi-fin design of heat sink and the finite element method to obtain the optimal configuration with an improved lifetime. Zhang et al. [7] carried out an optimal design and thermal simulation of the high-power LED array combined with the heat sink and microchannel. Xi and Schubert [8] presented the junction temperature of the GaN ultraviolet LED based on the proposed method using the temperature coefficient of the forward voltage. Lee et al. [9] also estimated the junction temperature based on a measurement of the thermal resistance employing a micro-temperature sensor as a real-time monitoring method for the junction temperatures. Keppens et al. [10] present measurement results of the current-voltage characteristics in commercial high-power LEDs. The measured results are modeled in terms of variation in the bandgap with temperature and show a linear temperature dependence of their forward voltages when the drive current is applied within the pre-determined current range. Even though the voltage intercept can be deduced from the bandgap, the accurate junction temperature is obtained only if two or more calibration measurements under a specific drive current are conducted. Alshahed et al. [11] present the compact thermal model extraction of package based on the measurement using their test chip with multiple heating and elements for sensing temperature to estimate the distribution of temperature in packaged chips. They showed that ultrathin semiconductors below 20 μm dramatically increased temperature, and they successfully measured in the time and frequency domain. The results showed that the temperature variations were strongly dependent on the frequency of heat pulsating. Chen et al. [12] explored the reason for the higher measurement than the phosphor temperature. They inserted the bead of the thermocouple into the phosphor layer, and they found that the thermocouple reveals a much higher temperature than the actual phosphor temperature due to the light energy absorption of the thermocouple corresponds to 20% of optical power at the supplied current of 350 mA. The over-estimated temperature of thermocouples causes severe temperature measurement error for the luminous devices and is highly related to the absorption coefficient of its material which has different values along with the wavelength [13]. Figure 1 shows light absorption ratios of typical metal materials.

The energy and optical efficiency of the lighting application using luminous devices also have been researched to enhance their advantages on green technology. Wang et al. investigate a design of aluminum nitride (AlN) based substrate for high-power LED to improve heat dissipation and its efficiency of multi-chip LED by taking advantage from hybrid substrate design, such as simple assembly on a printed circuit board, providing the subsidiary protection of ceramic and decrease of production cost [14]. Lee et al. [15] present a phosphor-in-glass (PIG) material with characteristics of high efficiency and conductance, which is employed as an optical element for white conversion in laser-diode (LD)-driven white-light systems. Their optimized PIG showed promising performance with lower component temperature and high efficiency in white-light extraction. Park et al. [16] present the experiment result of enhanced light extraction material of the LED. They applied ZrO_2_ nanoparticles of average particle size with 10 nm to the adhesive layer as an enhancer of refractive index and light scattering additives to increase the luminous efficacy and luminous flux of the white-LED in the form of polymer matrix nanocomposites.

In this study, a new type of photo-thermal sensor is introduced, aiming low-cost qualification in pilot line for high-power luminous devices and optical monitoring of costly luminaire such as automobile LED headlamp, and a compact method of both optical power and temperature measurement is achieved by employing the photo-thermal sensor. The principle of the introduced photo-thermal sensor is based on photo-absorbent heat from the optical power of the luminous device. The photo-thermal sensor consists of a couple of sheet-type thermocouples which can measure the temperature increase from the transferred thermal energy and absorbed optical energy on the hot spot of the luminous devices. Because two sheet-type thermocouples are designed to have the same thermal property and different optical absorbance coefficients, measured temperature responses of each sensor provide temperature difference which contains information of actual optical energy term. This net temperature between two sheet-type thermocouples is utilized as an output of a photo-thermal sensor which can measure the optical power and estimate the actual hot spot temperature of the luminous device. The proposed measuring method is evaluated by the measured optical power using the commercial integrating sphere and the actual temperature of the LED hot spot from an attached thermocouple.

## 2. Sensor Design

### 2.1. Photo–Thermal Sensor

The temperature measurement of the LED is significantly affected by the optical power absorption, and it leads to over-estimated temperature originated from additional thermal power input of the absorbed optical power. Eventually, the measurement error from light absorption makes it harder to manage the thermal system of the LEDs. To reduce the over-estimating error in indirect measurement and achieve the optical power measurement for the thermal management, the novel concept of the photo-thermal sensor is introduced with a couple of same sheet-type thermocouples that have a different light absorption coefficient each other. The difference of responses between two thermocouples is utilized as the sensor output of the photo-thermal sensor, which can present optical power history.

**Figure 2:**
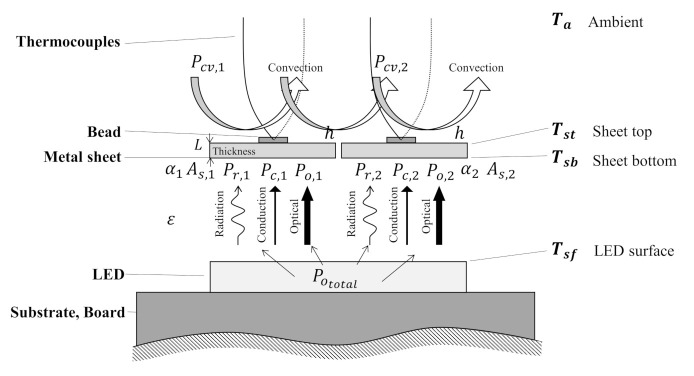
Schematics of the photo-thermal sensor.

The configuration of a photo-thermal sensor is depicted in Figure 2. In the figure, L, $A_{s}$, ε, k, and h denote the thickness, surface area, emissivity, thermal conductivity, and convective heat transfer coefficient of the metal sheet under the ambient temperature of $T_{a}.$ Moreover, the temperatures are denoted as $T_{sf}$ for the LED surface, $T_{st}$ for the top surface of the sheet, and $T_{sb}$ for the bottom surface. The sensor is composed of two radiative thermocouples, consisting of a thin metal sheet and a K-type thermocouple for each one. The metal sheet is utilized to quantify the absorbed optical power, which can be obtained by the physical calibration with the actual optical power output of the LED which is denoted as $P_{o_{total}}$.

As shown in Figure 2, each thermocouple experiences four major thermal power transfers, conduction, convection, radiation, and the direct absorption of optical power which are denoted as $P_{c}$, $P_{cv}$, $P_{r},$ and $P_{o}$. Each number in the subscription indicates the sensor number of sheet-type thermocouple. In the heat equation, however, the conduction through the air medium and radiation term are dependent $T_{sf}$ which is one of the measurement parameters. Therefore, the photo-thermal sensor is composed of a couple of thermocouple sensors to eliminate the variable of LED surface temperature from the sensor heat equation. The LED surface temperature can be removed by subtracting the temperature response of two thermocouples that have the same thermal property, only having different light absorption coefficients α. The output difference between two thermocouples is a key signal of the proposed photo-thermal sensor, which is intentionally provided by modifying the light absorption coefficient of a single thermocouple with a black dot on the metal sheet.

### 2.2. Heat Equation of the Photo–Thermal Sensor

The implementation of the photo-thermal sensor is based on the assumption that the sensor can be modeled as a lumped system accompanying the light absorption term. The dynamic thermal model with the lumped capacitance [17] is employed to derive the governing equation of the photo-thermal sensor under the following Biot number criterion.

(1) $$\frac{R_{cond}}{R_{conv}}=\frac{L/kA_{s}}{1/hA_{s}}=\frac{hL}{k}=Bi\ll 1$$

where, $A_{s}$ is the surface area of the metal sheet, $R_{cond}$ and $R_{conv}$ are the conductive and convective thermal resistances. 

The dimensional and thermal variables of the photo-thermal sensor are presented in Table 1. The aluminum is adopted as a material of metal sheet because of its high thermal conductivity with a thin thickness which satisfies the Biot number criterion of $\;7.9\times 10^{-7}$ and the assumption of representative sensing temperature denoted as $T_{s}$ of Equation (2).

**Figure 3:**
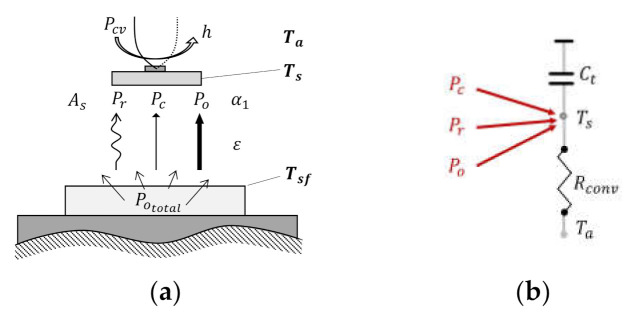
The lumped capacitance model of a single sheet-type thermocouple: (**a**) single thermocouple; (**b**) simplified thermal lumped capacitance model.

The thermal model of a single thermocouple in Figure 3a can be simplified into lumped capacitance model of Figure 3b with an assumption of representative sensing temperature and energy equation of lumped capacitance model using conductive, radiative, and photo-thermal heat input and convective heat dissipation through the ambient as follows:

(2) $$T_{s}\approx T_{sb}=T_{st}$$

(3) $$\frac{dE}{dt}=C_{t}\frac{dT_{s}}{dt}={\dot{E}}_{in}-{\dot{E}}_{out}+{\dot{E}}_{gen}=P_{c}+P_{r}+P_{o}+P_{cv}\;$$

(4) $$\begin{array}{c} C_{t}\frac{dT_{s}}{dt}=\frac{1}{R_{sf-s}}\left(T_{sf}-T_{s}\right)+F_{sf-s}\varepsilon A_{s}\sigma \left(T_{sf}^{4}-T_{s}^{4}\right)+F_{sf-s}\alpha P_{o_{total}}\\ +hA_{s}\left(T_{s}-T_{a}\right) \end{array}$$

where the σ, $C_{t},$ and $R_{sf-s}$ denote Stefan–Boltzmann constant, heat capacitance, and thermal resistance between the LED surface and metal sheet of the lumped model.

**Figure A1:**
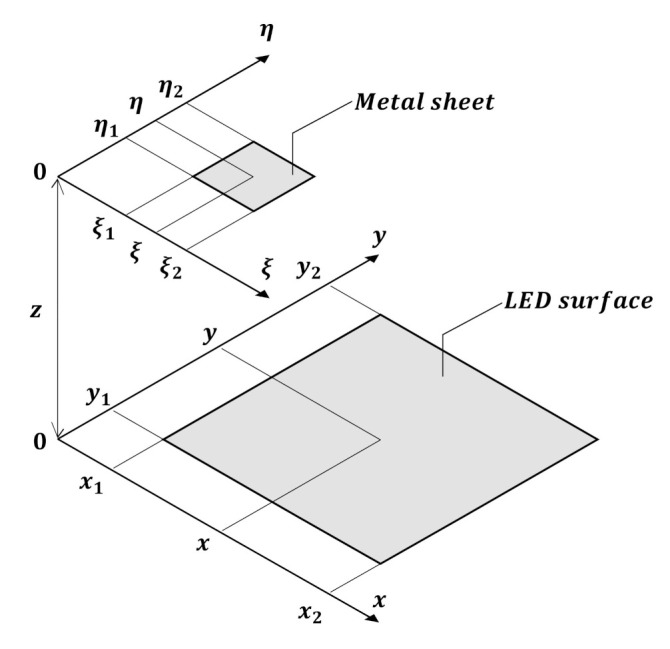
The geometric configuration between the LED surface and the metal sheet.

Radiation term can be linearized by introducing characteristic temperature, $T_{char}=\left(T_{s,1}+T_{s,2}\right)/2$ and temperature difference, $T_{net}=T_{s,1}-T_{s,2}$ as described. The view factor of the LED surface to the metal sheet denoted as $F_{sf-s}$ is also introduced to consider the geometric effect of both the radiative heat transfer and the light absorption. The heat input change caused by both the sensor location and the surface area difference can be implemented to heat equation by adopting the Equations (A1) and (A2) of view factor expression for arbitrary sized finite rectangles in the aligned parallel configuration of Figure A1 (Appendix A) [18].

(5) $$\begin{array}{c} C_{t}\frac{dT_{s,1}}{dt}=\frac{1}{R_{sf-s}}\left(T_{sf}-T_{s,1}\right)+F_{sf-s}\varepsilon A_{s}\sigma \left(T_{sf}^{4}-T_{s,1}^{4}\right)+F_{sf-s}\alpha_{1}P_{o_{total}}\\ +hA_{s}\left(T_{s,1}-T_{a}\right) \end{array}$$

(6) $$\begin{array}{c} C_{t}\frac{dT_{s,2}}{dt}=\frac{1}{R_{sf-s}}\left(T_{sf}-T_{s,2}\right)+F_{sf-s}\varepsilon A_{s}\sigma \left(T_{sf}^{4}-T_{s,2}^{4}\right)+F_{sf-s}\alpha_{2}P_{o_{total}}\\ +hA_{s}\left(T_{s,2}-T_{a}\right) \end{array}$$

As a result, the entire heat equation for a photo-thermal sensor is derived by subtracting Equation (6) from Equation (5) based on the assumption that two thermocouples have the same thermal properties and boundaries except for the light absorption coefficient as $\alpha_{1}$ and $\alpha_{2}$

(7) $$\begin{array}{c} C_{t}\frac{d}{dt}\left(T_{s,1}-T_{s,2}\right)+\frac{1}{R_{sf-s}}\left(T_{s,1}-T_{s,2}\right)+hA_{s}\left(T_{s,1}-T_{s,2}\right)\\ +F_{sf-s}\varepsilon A_{s}\sigma \left(T_{s,1}^{4}-T_{s,2}^{4}\right)\\ =F_{sf-s}\left(\alpha_{1}-\alpha_{2}\right)P_{o_{total}}\; \end{array}$$

(8) $$\begin{array}{c} C_{t}\frac{d}{dt}T_{net}+\frac{1}{R_{sf-s}}T_{net}+hA_{s}T_{net}+F_{sf-s}\varepsilon A_{s}\sigma \cdot 4T_{char}^{3}T_{net}\\ =F_{sf-s}\left(\alpha_{1}-\alpha_{2}\right)P_{o_{total}} \end{array}$$

where $T_{net}$ denotes temperature difference between sheet-type thermocouples.

## 3. Experiment and Results

### 3.1. Experimental Setup

**Figure 4:**
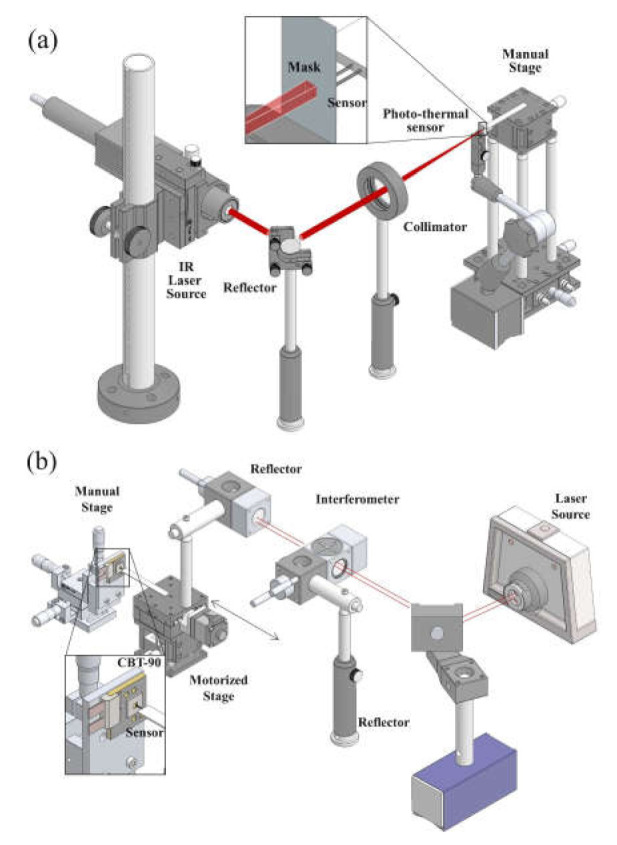
Experimental setup: (**a**) direct heat input test; (**b**) sensor response test setup.

The three different experiments on the photo-thermal sensor were carried out to characterize the thermal behavior of each thermocouple and to evaluate the performance of its measurement on the optical power and the LED surface temperature. Figure 4 illustrates the experimental setups for the photo-thermal sensor, and each setup aims to the validation of assumptions for the sensor heat equation. To produce the correct temperature difference between sheet-type thermocouple, as described in Equations (7) and (8), the photo-thermal sensor should be satisfied with the assumption that the two radiative type thermocouples show the same temperature response under the same direct heat input condition without the modification of light absorption coefficient.

In Figure 4a, the infrared (IR) laser source and optical parts in the experimental setup were utilized to liberate the heat directly to the metal sheet of each sensor. The metal mask with the 1$\;{\mathrm{mm}}^{2}$ size of the squared hole was also placed between the IR laser source and metal sheet to prevent the additional heat liberation from the LED to the photo-thermal sensor.

**Figure 5:**
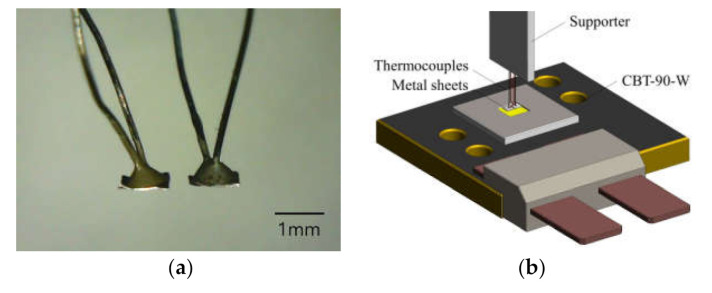
Configuration of sensor and measurement: (**a**) fabricated photo-thermal sensor; (**b**) photo-thermal measurement.

In the sensor response test, as shown in Figure 4b, the steady-state temperature response measurement along the sensor location from the LED was carried out to investigate the response of thermocouples influenced by the measuring distance. The same experimental setup was also employed to obtain the real-time sensor response under the stepped increase of the LED input power. Each result from these experiment setups was used to evaluate the effect of sensor distance on heat equation and to derive the sensitivity of the introduced photo-thermal optical power sensor. The evaluation of the sensor was performed by comparing the sensor response of step power input with measured optical power from the commercial integrating sphere (CAS 140CT, Instrument Systems, Munich, Germany). The commercial high power white light LED package (CBT-90-W, Luminus, Sunnyvale, USA) of Table 2 was employed in the experiments to investigate the thermal behavior of the introduced photo-thermal sensor and carry out its evaluation [19]. Each single thermocouple was fabricated by a pre-calibrated K-type thermocouple and a thin metal sheet of 10 μm thickness with 1$\;{\mathrm{mm}}^{2}$ of the area size containing 99.9% of aluminum, as shown in Figure 5a. Moreover, Figure 5b depicts the detailed configuration of the setup for photo-thermal measurement. A pair of the sheet-type thermocouples are located above the phosphor of the high-power LED, and temperature responses of each thermocouple are obtained independently during the measurement to evaluate the photo-thermal behavior.

### 3.2. Result and Discussion

**Figure 6:**
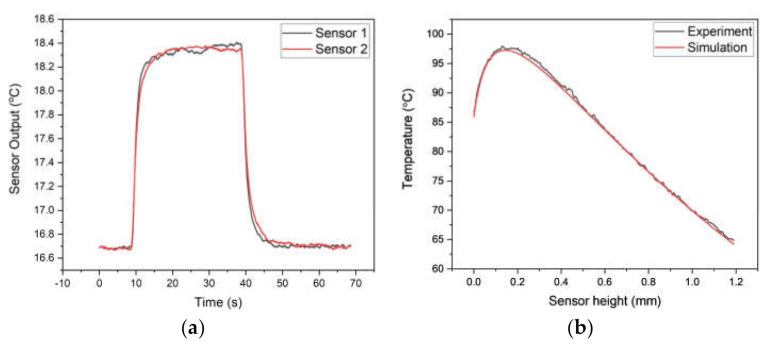
Sensor response results: (**a**) direct heating test; (**b**) sensor distance test.

As described in Equations (7) and (8), the introduced photo-thermal sensor employs thermal behavior of temperature response in thermocouple during the measurement, which is originated from the exchange of thermal energy between the sensor and the LED surface. The measurement of optical power is based on the difference in sensor response between thermocouples resulting from the difference in thermal input caused by the amount of light absorption only. Figure 6a compares the sensor response upon the direct heating experiment using two thermocouples without modification of light absorption. The responses show almost the same results within a maximum error of 0.7%, which indicates that the difference in thermal property between sensors can be ignored.

The introduced photo-thermal sensor also shows a unique concave steady-state temperature distribution along with the sensor distance. As described in the derivation of the heat equation, it can be explained that the distribution is originated from the changes in thermal resistance ($R_{sf-s}$) and optical power density, determined by the geometric configuration between the sensor and the LED. The view factor is introduced as Equation (A1) to implement these features to the heat equation. The measurement of steady-state temperature distribution along the sensor location was carried out by utilizing a precision stage shown in Figure 4b and comparing it with simulation results from the heat equation. As a result, the experiment and the analysis results of the single thermocouple showed almost identical results with unique temperature change. The maximum temperature deviation between the experiment and analysis results was 1.1 K at 0.5 mm, indicating that the introduced heat equation of the sensor can simulate the change of temperature response along with the sensor location within the maximum error of 1.3%, as shown in Figure 6b. In this study, the sensor was located at a distance of 0.5 mm from the LED surface.

**Figure 7:**
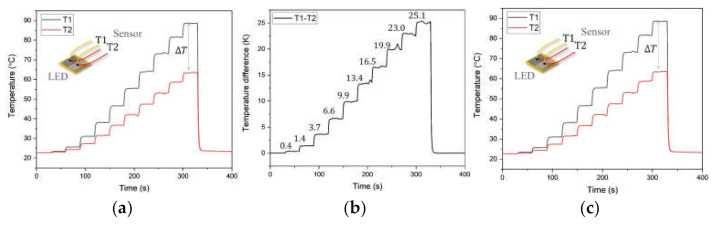
Characterization results: (**a**) temperature responses; (**b**) photo-thermal sensor response; (**c**) calibration result.

**Figure 8:**
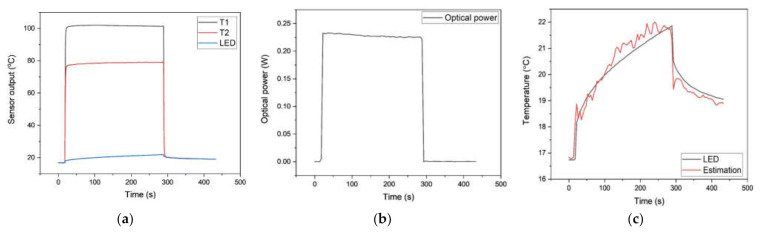
Measured temperature and optical power under the step power input of 1.8 W: (**a**) apparent temperature readings; (**b**) measured optical power; (**c**) estimated surface temperature of the LED.

**Figure 9:**
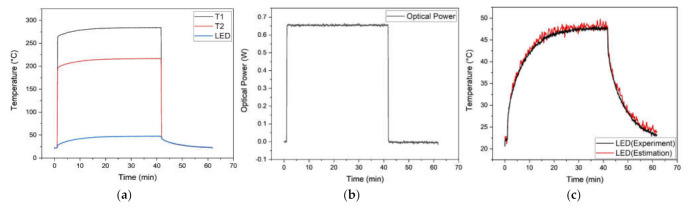
Measured temperature and optical power under the step power input of 5.5W: (**a**) apparent temperature readings; (**b**) measured optical power; (**c**) estimated surface temperature of the LED.

Figure 7, Figure 8 and Figure 9 present the experimental results of the optical power and surface temperature measurements of the LED for the evaluation of the proposed photo-thermal sensor. Figure 7a,b describe the temperature measurement results of thermocouples and photo-thermal sensor response under the stepped power input change for the sensor calibration. The sensor response was obtained during the input power is changed in 10 steps from 0.03 to 2.06 W, and the regression analysis was carried out to derive the physical relation between the response of the photo-thermal sensor and the measured optical power from the integrating sphere. In Figure 7c, $a_{s}$ and $b_{s}$ denote the calibration factor and bias of photo-thermal sensor, and the calibration result shows good linearity between the sensor response and measured optical power with 0.996 of $R^{2}$ as shown in the figure. This calibration result was utilized in the experiments to evaluate the proposed photo-thermal sensor in Figure 8 and Figure 9.

The evaluation of the proposed photo-thermal sensor was carried out based on the comparisons of the optical power measurement and the LED surface temperature estimation from a photo-thermal sensor with the pre-measured optical power and surface temperature result from an integrating sphere and attached thermocouple by the junction. As shown in Figure 8a and Figure 9a, each thermocouple depicts an overestimated temperature result caused by light absorption under the step input power conditions of 1.8 W and 5.5 W. The measurement of optical power is also performed by utilizing the photo-thermal sensor based on the calibration result from Figure 7c. The pre-measured optical power from the integrating sphere was 223.8 and 645.2 mW for 1.8 and 5.5 W of inputs, respectively. As presented in Figure 8b and Figure 9b, the real-time optical power measurement employing the proposed sensor shows a good result with 3.9% and 3% of maximum errors. Figure 8c and Figure 9c show the comparison result between the estimated and measured surface temperature of the LED. The estimated LED surface temperature was also obtained through the Figure 4a for a single thermocouple by utilizing the measured optical power and the temperature responses of Figure 8a,b and Figure 9a,b in a real-time fashion.

## 4. Conclusions

In this study, the compact measurements of the LED optical power and the hot spot temperature of the LED are introduced by employing a photo-thermal sensor based on the photo-absorbent power of the thermocouple. For this purpose, a pair of sheet-type thermocouples are designed and fabricated to compose a photo-thermal sensor realizing real-time measurement with the simplified sensor equation and minimized ambient effect. The modeling of the sensor equation is established based on the lumped capacitance model of each sheet-type thermocouples with consideration of the view factor and additional optical power absorption term. The fabricated photo-thermal sensor was calibrated and evaluated by measuring the optical power and the surface temperature of the LED. From the results, the following achievements are obtained.

First, the response of stepped input power shows high linearity with the regression $R^{2}$ value of 0.996 with the predetermined optical power of LED. Second, the optical power measurement and surface temperature estimation produce good results with the maximum error of 3.9% (9.2 mW) and 3% (11 mW) for 1.8 and 5.5 W of step input powers and maximum differences in surface temperature estimation as 1.1 and 3.2 K for each input power cases. The evaluation result indicates that the introduced photo-thermal sensor is expected to be capable of a novel, compact measurement method for the crucial performances of LED applications in a real-time fashion [20].

## Figures and Tables

**Table 1:** Dimensional and thermal properties of the sensor.

**Variables**	$A_{s}$	L	k	h
Value	1 mm^2^	10 μm	205 W/(mK)	6.73 W/(m^2^K)

**Table 2:** Dimensional and thermal properties of the sensor.

Forward Voltage Range	Operating Current Range	Emitting Area	Chip Type
2.9–3.8 V	0.2–18 A	$9{\;\mathrm{mm}}^{2}$	COB

**Figure A2:**
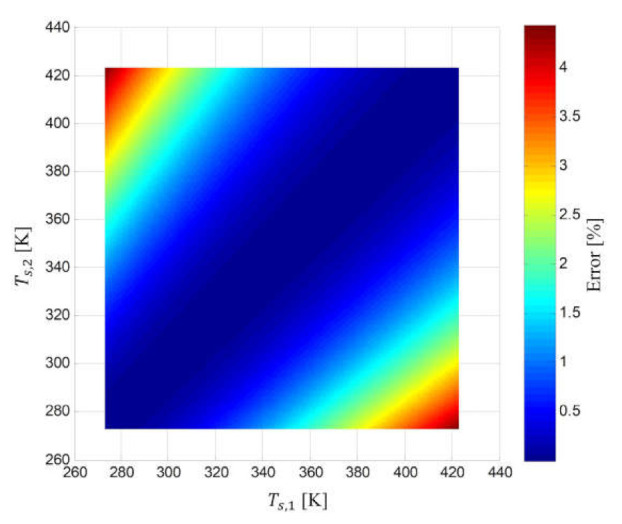
Error distribution of linearized radiation term (273–423 K).

## Appendix A. View Factor Calculation between Two Parallel Surfaces

(A1) $$F_{sf-s}=\frac{1}{\left(x_{2}-x_{1}\right)\left(y_{2}-y_{1}\right)}\overset{2}{\underset{l=1}{\sum }}\overset{2}{\underset{k=1}{\sum }}\overset{2}{\underset{j=1}{\sum }}\overset{2}{\underset{i=1}{\sum }}{\left(-1\right)}^{\left(i+j+k+l\right)}G\left(x_{i},y_{j},\eta_{k},\xi_{l}\right)\;$$

(A2) $$G=\frac{1}{2\pi }\left(\begin{array}{c} \left(y-\eta \right)\sqrt{{\left(x-\xi \right)}^{2}+z^{2}}\tan^{-1}\left\{\frac{y-\eta }{\sqrt{{\left(x-\eta \right)}^{2}+z^{2}}}\right\}\;\\ +\left(x-\xi \right)\sqrt{{\left(y-\eta \right)}^{2}+z^{2}}\tan^{-1}\left\{\frac{x-\xi }{\sqrt{{\left(y-\eta \right)}^{2}+z^{2}}}\right\}\\ -\frac{z^{2}}{2}\ln\left[{\left(x-\xi \right)}^{2}+{\left(y-\eta \right)}^{2}+z^{2}\right] \end{array}\right)$$

where x, y ζ, η are coordinates of each point in the metal sheet and LED surface area with the distance between the LED and metal sheet denoted as z.

## Appendix B. Linearization of Radiation Term

(A3) $$\begin{array}{ll} F_{sf-s}\varepsilon A_{s}\sigma \left(T_{s,1}^{4}-T_{s,2}^{4}\right) & =F_{sf-s}\varepsilon A_{s}\sigma \left(T_{s,1}^{2}+T_{s,2}^{2}\right)\left(T_{s,1}+T_{s,2}\right)\left(T_{s,1}-T_{s,2}\right)\\ & =F_{sf-s}\varepsilon A_{s}\sigma \left(2T_{char}^{2}\right)\left(2T_{char}\right)\left(T_{s,1}-T_{s,2}\right)\\ & =F_{sf-s}\varepsilon A_{s}\sigma \cdot 4T_{char}^{3}\cdot T_{net} \end{array}$$

(A4) $$\;T_{char}=\frac{T_{s,1}+T_{s,2}}{2}$$

Figure A2 depicts the simulated error distribution of the linearized radiation term within the range from 273 to 423 K for each sensor. The result indicates that the linearized radiation term can be utilized within 2% of error for conventional temperature difference range.
